# Advancements in Surgical Management of Carpal Tunnel Syndrome: A Comparative Review of Open, Endoscopic, and Ultra‐Minimally Invasive Techniques

**DOI:** 10.1155/aort/9652002

**Published:** 2026-08-31

**Authors:** Nikeeta Nancoo, Ira Bhasin, Nasir Kharma, Yousef Al Shahomi, Amar Shamsah, Ezzeldin A. Shalaby, Amanda Jane Abel, Aaron Samuel, Helal Salem Alhajri, Najeeb Ansari, Bramaes Dahal, Manju Rai

**Affiliations:** ^1^ School of Medicine, St. George’s University, True Blue, Grenada, sgu.edu; ^2^ Kasturba Medical College, Mangalore, India, kmc-mangalore; ^3^ King’s College Hospital NHS Trust, London, UK; ^4^ School of Medicine and Dentistry, University of Lancashire, Preston, UK; ^5^ University of Calgary, Calgary, Alberta, Canada, ucalgary.ca; ^6^ Faculty of Medicine, Alexandria University, Alexandria, Egypt, alexu.edu.eg; ^7^ Perdana University-Royal College Surgeons of Ireland, Kuala Lumpur, Malaysia; ^8^ Georgian National University SEU, Tbilisi, Georgia; ^9^ College of Medicine and Health Sciences, UAE University, Alain, UAE, uaeu.ac.ae; ^10^ Texila American University, Georgetown, Guyana, tauedu.org; ^11^ Kathmandu Medical College, Kathmandu, Nepal, kmc.edu.np; ^12^ Shri Venkateshwara University, Gajraula, Uttar Pradesh, India, svu.edu.in

**Keywords:** carpal tunnel syndrome, endoscopic carpal tunnel release, median nerve compression, minimally invasive surgery, open carpal tunnel release, ultrasound-guided carpal tunnel release

## Abstract

**Purpose:**

Carpal tunnel syndrome (CTS) is the most common entrapment neuropathy, with surgical decompression as the definitive treatment for refractory or severe cases. With the evolution of surgical techniques, from open carpal tunnel release (OCTR) to endoscopic (ECTR) and ultra‐minimally invasive ultrasound‐guided approaches, there remains a need for a comprehensive comparative evaluation. This review aims to critically assess contemporary surgical modalities for CTS and to provide an evidence‐based framework for patient‐centered surgical decision‐making.

**Methods:**

A structured narrative review was conducted using PubMed/MEDLINE, Scopus, Cochrane Library, and Web of Science, covering studies from 1985 to February 2026. Eligible studies included randomized controlled trials, cohort studies, systematic reviews, and meta‐analyses reporting clinical outcomes of OCTR, ECTR, and ultrasound‐guided techniques. Outcomes assessed included symptom relief, functional scores, complications, recurrence, return to work, and cost‐effectiveness. Data were synthesized thematically due to heterogeneity.

**Results:**

OCTR and ECTR demonstrate comparable long‐term symptom relief and functional outcomes. ECTR offers advantages in early postoperative recovery, including reduced pain and faster return to work. Ultra‐minimally invasive ultrasound‐guided techniques show promising early outcomes and accelerated recovery in selected patients, though long‐term data remain limited. Complication rates are low across techniques and are influenced more by surgical expertise and completeness of release than by approach alone. Economic analyses suggest early recovery may offset higher procedural costs in working populations.

**Conclusion:**

Long‐term outcomes of CTS surgery are broadly equivalent across techniques when adequate decompression is achieved. The surgical approach should be individualized based on disease severity, patient factors, occupational demands, and surgeon expertise. Further high‐quality, long‐term comparative studies are needed to define the role of emerging ultra‐minimally invasive techniques.

## 1. Introduction

Carpal tunnel syndrome (CTS) is the most prevalent entrapment neuropathy worldwide and accounts for approximately 90% of peripheral nerve compression syndromes [1]. Population‐based studies estimate a prevalence of 3%–5% in the general population, with higher incidence among women, middle‐aged individuals, and patients engaged in repetitive manual occupations [1, 2]. Beyond its clinical manifestations, CTS imposes a substantial socioeconomic burden due to work absenteeism, reduced productivity, and healthcare resource utilization [2]. Pathophysiologically, compression of the median nerve within the carpal tunnel results in nocturnal paresthesia, pain, numbness, and, in advanced stages, thenar weakness and muscle atrophy [3].

CTS is multifactorial in origin. Occupational exposure to repetitive wrist motion, forceful gripping, vibration, and sustained wrist flexion increases intracarpal pressure and predisposes to median nerve compression [2]. Individual risk factors, including obesity, diabetes mellitus, hypothyroidism, pregnancy, inflammatory arthropathies, and anatomical variations, further contribute to disease susceptibility and severity [3, 4]. The wide demographic and etiologic spectrum of CTS underscores the need for individualized treatment strategies that account for both systemic and mechanical contributors.

Conservative management remains the first‐line treatment for mild to moderate CTS and includes splinting, pharmacologic therapy, corticosteroid injections, and rehabilitative interventions [5]. Although these modalities may provide short‐term symptom relief, high‐quality systematic reviews demonstrate that surgical decompression offers superior long‐term symptom resolution, functional recovery, and electrophysiological improvement in patients with persistent or severe disease [6, 7]. As a result, surgical release of the transverse carpal ligament (TCL) has become the definitive treatment for refractory CTS.

Over the past several decades, surgical management has evolved from traditional open carpal tunnel release (OCTR) to endoscopic carpal tunnel release (ECTR), and more recently to ultra‐minimally invasive ultrasound‐guided and percutaneous techniques [7, 8]. While long‐term clinical outcomes appear broadly comparable among established techniques, differences in early postoperative recovery, complication profiles, cost‐effectiveness, and learning curves continue to influence procedural selection [8]. With ongoing technological advancements and increasing emphasis on patient‐centered care, a contemporary comparative evaluation of surgical approaches is warranted.

Despite multiple systematic reviews and meta‐analyses comparing OCTR and ECTR, important gaps remain in translating this evidence into individualized clinical decision‐making. Existing evidence syntheses primarily focus on pairwise comparisons of surgical techniques, whereas contemporary practice increasingly incorporates patient‐specific factors, surgeon expertise, economic considerations, and emerging ultrasound‐guided ultra‐minimally invasive procedures. Furthermore, recent technological advances and newer comparative studies have not yet been integrated into a unified clinical framework. Rather than merely summarizing available evidence, this narrative review critically appraises the strengths and limitations of contemporary surgical approaches, identifies persistent evidence gaps, and proposes a practical patient‐centered framework to guide surgical selection in routine clinical practice.

## 2. Literature Search and Narrative Synthesis Approach

A comprehensive literature search was conducted to identify studies evaluating the surgical management of CTS. Electronic databases including PubMed/MEDLINE, Scopus, Web of Science, and the Cochrane Library were searched for articles published between January 1985 and February 2026. The final search was performed on February 28, 2026.

The search strategy combined Medical Subject Headings (MeSH) and free‐text keywords using Boolean operators (AND/OR). The principal search terms included “carpal tunnel syndrome,” “median nerve compression,” “carpal tunnel release,” “open carpal tunnel release,” “endoscopic carpal tunnel release,” “ultrasound‐guided carpal tunnel release,” “ultra‐minimally invasive carpal tunnel release,” “percutaneous carpal tunnel release,” “thread carpal tunnel release,” “needle carpal tunnel release,” “minimally invasive surgery,” “surgical outcomes,” “complications,” and “functional recovery.” Database‐specific subject headings and syntax were adapted where appropriate.

Only articles published in English involving human subjects were considered. Reference lists of relevant systematic reviews, meta‐analyses, and key primary studies were also manually screened to identify additional eligible publications that were not retrieved through the electronic database search.

Studies were considered relevant for inclusion if they reported clinical outcomes following OCTR, ECTR, or ultrasound‐guided/percutaneous release in adult patients with clinically and/or electrophysiologically confirmed CTS. Relevant study designs included randomized controlled trials (RCTs), prospective and retrospective cohort studies, systematic reviews, and meta‐analyses. Studies were required to report at least one clinically relevant outcome, such as symptom relief, validated functional scores, including the Boston Carpal Tunnel Questionnaire (BCTQ) or Disabilities of the Arm, Shoulder and Hand score (DASH), complication rates, recurrence or reoperation rates, return‐to‐work timelines, or economic outcomes. Cadaveric investigations were selectively included when describing the technical feasibility of emerging procedures. Case reports, very small case series, and non‐English publications were excluded unless historically significant.

The literature search and initial identification of relevant articles were performed by the primary reviewers. Potentially eligible full‐text articles were subsequently evaluated against the predefined eligibility criteria. To minimize selection bias, the same eligibility criteria were applied consistently throughout the review process, and any uncertainties regarding study eligibility or data interpretation were resolved through discussion and consensus among the author team. Consistent with the methodology of a structured narrative review, duplicate independent screening of all retrieved records was not performed.

### 2.1. Risk of Bias and Quality Assessment

Because this review incorporated multiple study designs, a design‐appropriate approach to quality appraisal was adopted rather than applying a single uniform instrument. RCTs were appraised using the Cochrane Risk of Bias 2 (RoB 2) tool across the domains of randomization process, deviations from intended interventions, missing outcome data, measurement of the outcome, and selection of the reported result. Systematic reviews and meta‐analyses were appraised using AMSTAR‐2 (A MeaSurement Tool to Assess systematic Reviews), which evaluates protocol registration, adequacy of the literature search, duplicate study selection and data extraction, and management of risk of bias in the primary studies. Nonrandomized cohort and registry studies were appraised using the Newcastle–Ottawa Scale (NOS), which rates studies across the domains of selection, comparability, and outcome ascertainment. Preprint publications that had not undergone peer review were flagged as carrying indeterminate risk of bias and were not scored using a standardized tool, consistent with the inability to independently verify methodological conduct prior to peer review. Quality appraisal was performed by the primary reviewers, with disagreements resolved by discussion and consensus among the author team, consistent with the study selection process described above. Findings of this appraisal are summarized in Supporting Table S1 and were used to contextualize the strength of evidence underlying each thematic conclusion, with studies rated as high risk of bias or indeterminate quality interpreted with additional caution throughout the narrative synthesis.

Data were extracted narratively, with emphasis on study design, patient population characteristics, sample size, duration of follow‐up, outcome measures, complication profiles, recurrence rates, recovery timelines, and economic implications. Given substantial heterogeneity in technique definitions, surgeon experience, and outcome reporting across studies, a formal quantitative meta‐analysis was not performed. Instead, evidence was synthesized thematically across domains, including clinical outcomes, complication profiles, recovery and return to work, cost‐effectiveness, learning curve considerations, patient selection, and emerging technologies.

**FIGURE 1:**
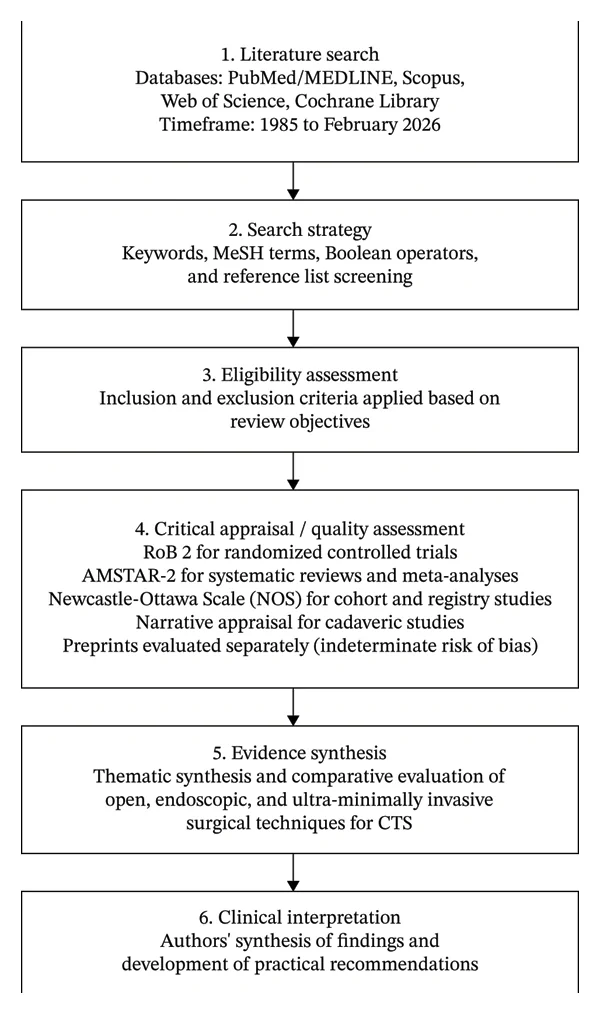
Overview of the literature search and article selection process undertaken for this narrative review. Abbreviation: CTS, carpal tunnel syndrome.

This review was conducted and is reported as a structured narrative review, following the Scale for the Assessment of Narrative Review Articles (SANRA) framework rather than the PRISMA Statement used for systematic reviews. A reproducible search strategy, predefined selection criteria, and design‐specific methodological quality appraisal were incorporated to enhance transparency and strengthen interpretation of the evidence. However, protocol registration, duplicate independent screening of all retrieved records, and quantitative meta‐analysis were not undertaken, consistent with the methodological framework of a structured narrative review. The overall methodological workflow, including the literature search, eligibility assessment, quality appraisal, evidence synthesis, and clinical interpretation, is illustrated in Figure 1.

## 3. Surgical Anatomy and Pathophysiology

**FIGURE 2:**
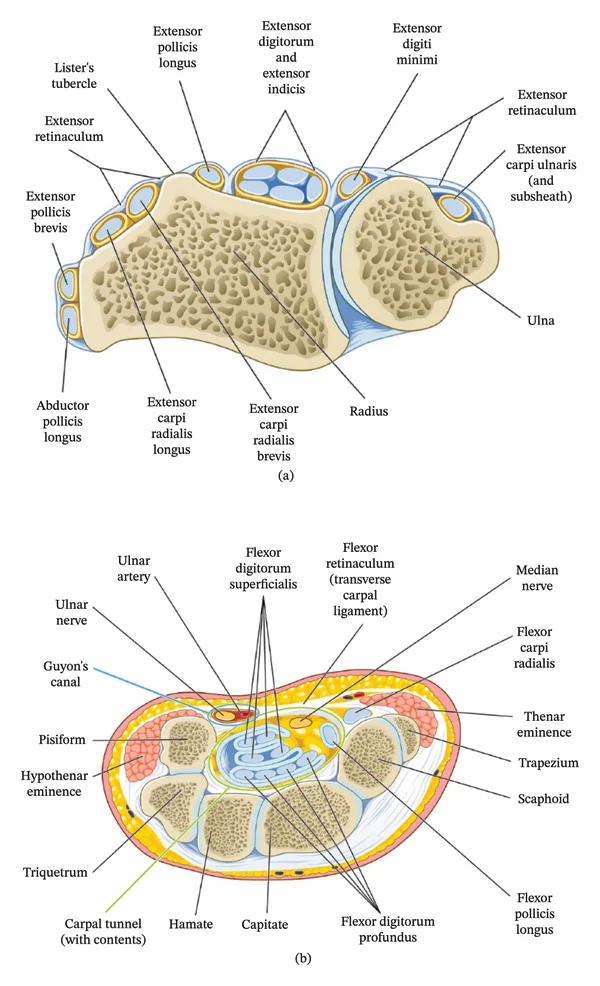
Cross‐sectional anatomy of the wrist: (a) Dorsal osteofibrous compartments and extensor tendons; (b) volar carpal tunnel, flexor retinaculum, median nerve, and associated neurovascular structures (this illustration was created by the author, Ira Bhasin, using the Biorender.com app).

The carpal tunnel is a fibro‐osseous canal located at the volar aspect of the wrist, bounded dorsally by the carpal bones and volarly by the TCL, also referred to as the flexor retinaculum [9]. Within this confined space course are the median nerve and nine flexor tendons (four flexor digitorum superficialis, four flexor digitorum profundus, and the flexor pollicis longus) (Figure 2). The median nerve typically lies superficial and slightly radial within the tunnel, rendering it particularly vulnerable to increases in intracarpal pressure [9]. Distally, the nerve divides into terminal motor and sensory branches, including the recurrent motor branch, whose variable course is of particular surgical relevance [10].

The pathophysiology of CTS centers on elevated intracarpal pressure leading to mechanical compression and ischemic insult of the median nerve. Sustained or repetitive wrist flexion and extension, synovial hypertrophy, fluid retention, and structural narrowing can increase tunnel pressure beyond the threshold tolerated by intraneural microcirculation [11]. This results in impaired axonal transport, segmental demyelination, and, in advanced stages, axonal loss [11, 12]. Early clinical manifestations, such as nocturnal paresthesia and intermittent numbness, correspond to reversible ischemia and demyelination, whereas persistent compression may culminate in thenar muscle weakness and atrophy due to motor fiber involvement [12].

Surgical decompression aims to reduce intracarpal pressure by dividing the TCL, thereby restoring neural perfusion and halting progressive nerve injury [13]. The adequacy of ligament release is critical; incomplete division remains a recognized cause of persistent or recurrent symptoms. Anatomic variations, including bifid median nerve, persistent median artery, anomalous motor branch course, and aberrant muscle bellies, may influence both surgical approach and complication risk [10, 14]. Awareness of these variants is particularly important in endoscopic and ultra‐minimally invasive techniques, where visualization may be limited.

Furthermore, the superficial palmar arch and recurrent motor branch are structures at risk during distal ligament division, emphasizing the need for precise identification of distal release limits [14]. Understanding these anatomical relationships not only informs surgical planning but also underpins technique selection, complication avoidance, and optimization of clinical outcomes across open, endoscopic, and ultrasound‐guided approaches.

## 4. Indications for Surgical Intervention

Surgical intervention for CTS is primarily indicated in patients who demonstrate persistent symptoms despite an adequate trial of conservative management. First‐line therapies, including splinting, pharmacologic agents, corticosteroid injections, and structured rehabilitation, are generally recommended for mild to moderate CTS [15]. Recent evidence has also suggested that shear wave elastography may provide objective information on median nerve stiffness changes after ultrasound‐guided corticosteroid injection and may therefore contribute to treatment–response assessment and individualized decision‐making in patients with CTS [16].

However, failure of conservative treatment is characterized by ongoing pain, paresthesia, nocturnal symptoms, or functional impairment after an appropriate duration of therapy, typically 6–12 weeks, or recurrence of symptoms following temporary relief from injections [17]. In such cases, surgical decompression of the TCL is associated with superior long‐term symptom resolution and functional recovery compared with continued nonoperative management [17, 18].

Severity‐based indications further guide operative decision‐making. Clinically, patients presenting with constant numbness, progressive weakness, impaired dexterity, or thenar muscle atrophy warrant prompt surgical evaluation. Electrophysiological studies demonstrating moderate to severe median neuropathy, characterized by prolonged distal motor latency, reduced sensory nerve conduction velocity, or evidence of axonal loss, are strong predictors of poor response to conservative measures and support early decompression [19]. Importantly, severe CTS with evidence of denervation on electromyography is considered an absolute indication for surgical release to prevent irreversible motor deficit [19, 20].

Certain special populations require individualized consideration. Patients with diabetes mellitus often present with more severe symptoms and may have concomitant peripheral neuropathy; although surgical outcomes are generally favorable, recovery may be slower and complication rates slightly higher, necessitating careful perioperative optimization [21]. In pregnancy, CTS is frequently related to fluid retention and hormonal changes, with many cases resolving postpartum; therefore, surgery is typically reserved for severe, refractory cases or those with progressive neurological deficit [22]. Elderly patients may benefit significantly from decompression, although comorbidities and anesthetic risk must be evaluated on an individual basis [20]. Similarly, individuals with occupational CTS, particularly those engaged in repetitive or forceful manual labor, may require earlier surgical consideration when symptoms compromise work capacity or safety [15].

Overall, indications for surgery in CTS integrate symptom persistence, clinical severity, electrophysiological findings, and patient‐specific factors. A structured, individualized approach ensures timely intervention while minimizing unnecessary operative risk.

## 5. Historical Evolution of Surgical Techniques for CTS

Surgical management of CTS has undergone progressive refinement over the past century, driven by the dual objectives of achieving reliable median nerve decompression while minimizing morbidity. Early descriptions of surgical release date to the mid‐20th century, when open division of the TCL emerged as a definitive intervention for refractory CTS [23]. Phalen’s landmark clinical series established OCTR as the standard approach, demonstrating durable symptom resolution with direct visualization of the median nerve and surrounding structures [23]. For decades, OCTR remained the gold standard owing to its reproducibility and low recurrence rates.

However, despite its efficacy, traditional open techniques were associated with wound‐related morbidity, pillar pain, and delayed return to work. As functional outcome measures became increasingly emphasized in the late 20th century, efforts shifted toward minimizing surgical trauma without compromising decompression adequacy. This led to the development of ECTR, first popularized in the late 1980s and early 1990s [24]. Early randomized trials comparing OCTR and ECTR demonstrated equivalent long‐term symptom relief but suggested faster early recovery and reduced postoperative pain with endoscopic approaches [25]. Nonetheless, these advantages were counterbalanced by concerns regarding neurovascular injury, higher procedural costs, and a steeper learning curve [26]. The evolution from open to endoscopic techniques thus reflected a trade‐off between visualization security and minimally invasive recovery benefits rather than unequivocal superiority.

More recently, advances in high‐resolution ultrasound and percutaneous instrumentation have enabled ultra‐minimally invasive approaches, including ultrasound‐guided carpal tunnel release (UGCTR). These techniques aim to further reduce incision size and soft tissue disruption while maintaining real‐time visualization of neural and vascular structures [27]. Early prospective and randomized studies suggest comparable short‐term functional outcomes relative to OCTR and ECTR, with potentially faster recovery profiles [28]. However, long‐term comparative data remain limited, and widespread adoption is contingent upon operator expertise and standardized outcome reporting.

Overall, the historical progression of CTS surgery reflects an iterative refinement process, moving from maximized visualization toward minimized tissue disruption, while maintaining the central therapeutic principle of complete TCL division. This evolution underscores the ongoing balance between surgical exposure, safety, recovery optimization, and technological innovation.

## 6. OCTR

### 6.1. Surgical Technique

OCTR remains the benchmark surgical technique for CTS. The standard approach involves a 2–4 cm longitudinal palmar incision aligned with the radial border of the ring finger, followed by careful dissection through subcutaneous tissue to expose and divide the TCL under direct visualization [29]. Complete ligament division is confirmed proximally and distally to ensure adequate decompression of the median nerve while protecting the recurrent motor branch and superficial palmar arch.

Mini‐open variations were developed to reduce incision length while preserving direct visualization. These approaches utilize smaller (1–2 cm) incisions and limited soft tissue dissection, maintaining the fundamental principle of full ligament release [30]. Unlike endoscopic or ultrasound‐guided techniques, OCTR allows unrestricted exposure, which is particularly advantageous in revision cases, space‐occupying lesions, or anomalous anatomy.

### 6.2. Advantages

The principal advantage of OCTR is direct visualization of the TCL and surrounding neurovascular structures. This visibility contributes to procedural reproducibility and a relatively short learning curve compared with endoscopic techniques [31]. For trainees and surgeons in lower‐volume settings, OCTR provides a reliable and standardized method with predictable outcomes.

Furthermore, OCTR demonstrates consistent long‐term efficacy across diverse patient populations, including complex presentations. Its durability and low recurrence rates have reinforced its role as the reference standard in comparative surgical studies.

### 6.3. Limitations and Complications

Despite its reliability, OCTR is associated with specific morbidities. Pillar pain and scar tenderness are reported in a subset of patients and may delay functional recovery [32]. Open techniques may also be associated with a longer time to return to work compared with minimally invasive approaches, particularly in manual laborers [33]. Rare but recognized complications include neurovascular injury, superficial palmar arch injury, and complex regional pain syndrome [32].

Reported recurrence rates after OCTR generally range from 1% to 5%, most commonly attributable to incomplete ligament division or postoperative fibrosis rather than technique failure per se [34].

### 6.4. Clinical Outcomes

High‐quality randomized trials and long‐term cohort studies demonstrate that OCTR provides durable symptom relief and sustained functional improvement in the majority of patients [35]. Long‐term follow‐up studies exceeding 10 years report stable symptom resolution and preserved grip strength, supporting its efficacy as a definitive intervention [34].

While minimally invasive approaches may offer advantages in early postoperative recovery, multiple systematic reviews confirm that long‐term functional outcomes between OCTR and alternative techniques are largely equivalent [30, 35]. Accordingly, OCTR remains the preferred option in complex cases requiring maximal visualization and continues to serve as the reference comparator for emerging techniques.

## 7. ECTR

### 7.1. Types of Endoscopic Techniques

ECTR represents a significant evolution in surgical management of CTS, aiming to minimize soft tissue trauma while maintaining effective median nerve decompression. Two principal approaches are described in contemporary literature: the single‐portal and dual‐portal techniques. The single‐portal approach, developed by Agee and colleagues, employs a single incision proximal to the wrist crease through which an endoscope and cutting instrument are introduced, allowing direct visualization of the ligament’s undersurface during division [36]. The dual‐portal technique, originally described by Chow, utilizes proximal and distal portals to permit simultaneous endoscopic illumination and instrument passage, theoretically enhancing visualization across the TCL [37]. Comparative evidence suggests similar clinical outcomes between these variants, with surgeon preference and familiarity driving technique selection.

### 7.2. Surgical Technique Overview

**FIGURE 3:**
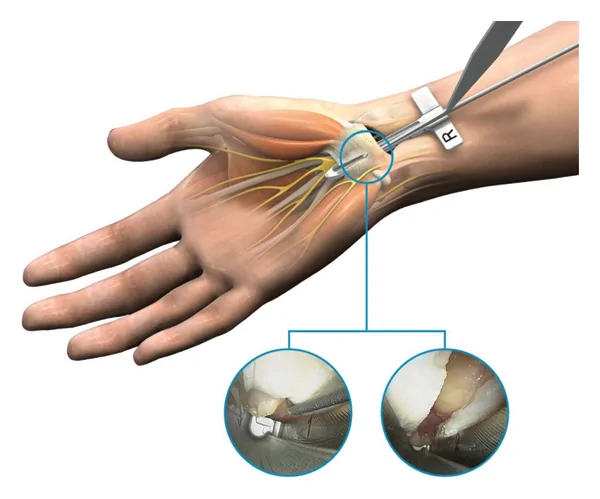
Endoscopic carpal tunnel release demonstrating cannula placement beneath the transverse carpal ligament. The wrist is positioned in neutral alignment, and an endoscopic cannula is introduced through a small proximal wrist incision. Under direct visualization, the transverse carpal ligament is divided from its undersurface while protecting the median nerve and adjacent neurovascular structures. Insets depict intraoperative endoscopic views of ligament transection and confirmation of complete release (this illustration was created by the author, Nikeeta Nancoo, using the BioRender.com app and incorporates original intraoperative endoscopic images from the author’s personal clinical collection).

In both single‐ and dual‐portal ECTR, the wrist is positioned in neutral or slight extension, and a 1–2 cm transverse or longitudinal incision is made at the wrist crease. After blunt dissection to the antebrachial fascia, the endoscopic cannula is introduced beneath the TCL (Figure 3). Under continuous visualization, a dedicated blade or cutting device is advanced to divide the ligament from distal to proximal, with care taken to identify and protect the recurrent motor branch and superficial palmar arch [38]. Modern endoscopic systems incorporate wide‐field optics and continuous irrigation to optimize visualization and reduce soft tissue friction.

### 7.3. Advantages

Recent randomized studies emphasize ECTR’s advantages in early postoperative recovery. A 2021 RCTs comparing ECTR with open release in a working population (*n* = 240) reported significantly less early postoperative pain (measured by VAS) and quicker resumption of manual work in the endoscopic group at 2 and 6 weeks postoperatively [39]. Functional outcomes, assessed by the BCTQ and grip strength, favored ECTR at 3 months but converged by 12 months. Meta‐analyses incorporating data up to 2024 corroborate these findings, indicating that ECTR is associated with reduced scar tenderness and earlier return‐to‐work without compromising long‐term relief [40].

### 7.4. Limitations and Complications

Despite its advantages, ECTR carries unique risks. Surgeon experience remains a critical determinant: in a large prospective cohort (*n* = 512), the rate of incomplete release was higher during the early learning curve, highlighting the need for structured training and volume thresholds [41]. Neurovascular injury, including injury to the median nerve or its motor branch, has been reported in 1%–3% of cases, with many occurring during initial adoption phases [41]. Additionally, equipment costs and operative time remain modestly higher than for open techniques, potentially limiting access in resource‐constrained settings.

### 7.5. Clinical Outcomes

Long‐term outcomes following ECTR are well characterized. A multicenter prospective study (*n* = 378) with 5‐year follow‐up demonstrated sustained symptom relief (BCTQ) improvement (≥ 50%) and comparable recurrence rates (< 5%) to open release [42]. Systematic reviews confirm that while early recovery metrics (pain, time to work) favor ECTR, measures such as long‐term functional status, grip strength, and electrophysiologic improvement do not differ significantly from OCTR [40]. Patient satisfaction surveys consistently rate ECTR highly, particularly in populations prioritizing rapid return to routine activities and minimal scarring.

In sum, ECTR offers a tissue‐sparing alternative to open release with demonstrable early benefits and equivalent long‐term efficacy, though its technical demands and complication profile underscore the importance of surgeon experience in optimizing outcomes.

## 8. Ultra‐Minimally Invasive Techniques

### 8.1. UGCTR

UGCTR represents a paradigm shift toward percutaneous decompression under real‐time imaging. Rather than constituting a single operative method, UGCTR functions as an imaging platform that facilitates multiple techniques for TCL division while permitting continuous visualization of the median nerve, its motor branches, and adjacent vascular structures [43].

High‐resolution ultrasound enables dynamic mapping of the carpal tunnel, including identification of the recurrent motor branch and anatomical variants, theoretically reducing blind dissection. Prospective cohort studies published between 2022 and 2025 involving predominantly mild‐to‐moderate CTS patients (sample sizes 40–150) report significant improvements in BCTQ scores within 2–6 weeks and sustained symptom reduction at 12 months [44, 45]. However, these studies are largely single‐center and nonrandomized, limiting generalizability. Consequently, although UGCTR has demonstrated encouraging early clinical outcomes, its current role should be considered complementary to established surgical approaches and is presently best suited to carefully selected patients treated by surgeons experienced in musculoskeletal ultrasound.

### 8.2. Thread‐Based and Needle‐Based Release Techniques

**FIGURE 4:**
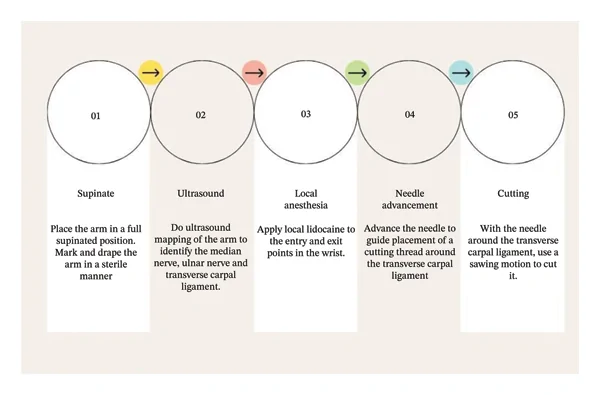
Stepwise schematic representation of ultrasound‐guided thread and needle‐based carpal tunnel release. The procedure includes wrist supination and sterile preparation, ultrasound mapping of the median nerve and transverse carpal ligament, administration of local anesthesia, guided advancement of a specialized needle beneath the transverse carpal ligament, and controlled deployment of a cutting mechanism to achieve ligament division and median nerve decompression (this illustration was created by the author, Najeeb Ansari, using the BioRender.com app).

Thread‐based release utilizes a percutaneously looped cutting filament placed circumferentially around the TCL under ultrasound guidance. Controlled reciprocal traction divides the ligament in a “sawing” motion (Figure 4). Recent pilot series and early clinical cohorts have reported technical success rates exceeding 95% [45, 46]; however, these findings are derived from relatively small, highly selected patient populations with limited follow‐up and should therefore be considered preliminary. Larger randomized comparative studies with long‐term follow‐up are required before these techniques can be considered equivalent to established OCTR or ECTR.

Needle‐based release employs a needle‐mounted blade or specialized microknife introduced under ultrasound guidance to divide the TCL incrementally. Early cadaveric validation studies confirmed feasibility and complete ligament sectioning [47]. Subsequent small clinical cohorts demonstrate rapid symptom relief and return to daily activities within 1–2 weeks, though robust long‐term comparative data remain sparse [48]. However, the safety, durability, and long‐term effectiveness of these techniques relative to established OCTR and ECTR have not yet been established.

### 8.3. Advantages

Ultra‐minimally invasive techniques are typically performed under local anesthesia in an outpatient setting. Reported advantages include minimal soft tissue disruption, reduced postoperative pain, and return to work within 7–14 days in selected populations [44, 46]. Cosmetic outcomes are favorable due to subcentimeter entry points. However, these reported benefits are derived primarily from early prospective studies involving carefully selected patients and experienced operators. Therefore, these findings should not be interpreted as demonstrating superiority or equivalence to established open or endoscopic techniques until validated in larger randomized comparative trials with long‐term follow‐up.

### 8.4. Limitations and Concerns

Despite promising early outcomes, several concerns persist. First, operator dependency is substantial; proficiency requires advanced musculoskeletal ultrasound training. Inadequate visualization may increase the risk of incomplete release or iatrogenic nerve injury. Second, patient selection is critical. Most published cohorts exclude patients with severe axonal loss, space‐occupying lesions, or significant anatomic variation [43, 45]. Third, high‐quality multicenter randomized trials comparing UGCTR directly with OCTR or ECTR remain limited.

Importantly, long‐term recurrence data beyond 5 years are currently unavailable. While short‐term complication rates appear low, the durability of decompression and long‐term recurrence rates remain uncertain. Accordingly, widespread adoption should be approached cautiously until high‐quality multicenter comparative studies with extended follow‐up establish the long‐term safety, effectiveness, and reproducibility of these techniques across diverse clinical settings.

### 8.5. Current Evidence and Early Outcomes

Current systematic reviews suggest that UGCTR offers early functional improvement and faster postoperative recovery compared with conventional techniques in carefully selected patients [43]. However, these findings should be interpreted cautiously. Much of the available evidence is derived from single‐center prospective studies, pilot trials, and highly selected patient populations treated by surgeons with advanced expertise in musculoskeletal ultrasound. Consequently, the reported benefits may reflect patient selection and operator experience as much as the intrinsic advantages of the technique itself. Furthermore, the absence of robust long‐term comparative data limits conclusions regarding durability, recurrence, and cost‐effectiveness. At present, UGCTR should therefore be regarded as a promising adjunct to established surgical techniques rather than a definitive replacement, pending confirmation through adequately powered multicenter randomized trials with standardized long‐term outcome reporting.

Key comparative clinical evidence evaluating open, endoscopic, and UGCTR techniques is summarized in Table 1. Formal risk‐of‐bias and quality appraisal of these key studies, performed using design‐appropriate instruments, is presented in Supporting Table S1; ratings ranged from high confidence for the Cochrane systematic review to indeterminate risk for the single non–peer‐reviewed preprint and were used to weight the strength of the narrative conclusions drawn below.

**TABLE 1:** Summary of key clinical studies comparing open, endoscopic, and ultrasound‐guided carpal tunnel release.

Author (year)	Study design	Level of evidence^∗^	*N*	Follow‐up	Key findings	Limitations
Peer‐reviewed clinical studies						
Agee et al., 1992 [36]	Multicenter randomized trial (ECTR vs. OCTR)	Level I (RCT)	147 hands	Up to 3 months	Earlier return to work and reduced scar tenderness with ECTR; similar symptom relief to OCTR.	Early‐generation endoscopic technology; limited long‐term PROMs.
Brown et al., 1993 [49]	Prospective randomized trial (ECTR vs. OCTR)	Level I (RCT)	169 hands	12 weeks	Earlier return to work and less scar tenderness after ECTR; comparable symptom relief.	Short follow‐up; early endoscopic instrumentation.
Sayegh & Strauch, 2015 [40]	Meta‐analysis of randomized controlled trials	Level I (meta‐analysis)	Pooled	Up to 12 months	ECTR associated with modest early recovery advantages; no clinically significant long‐term functional superiority over OCTR.	Heterogeneity among included trials; variability in surgeon experience.
Vasiliadis et al., 2014 [42]	Cochrane systematic review	Level I (systematic review)	Multiple RCTs	Variable	Confirms equivalent long‐term outcomes; ECTR improves early recovery and scar tenderness.	Variable methodological quality of included studies.
Louie et al., 2013 [50]	Retrospective cohort	Level III	157	> 10 years	Durable long‐term symptom relief, low reoperation rate, and high patient satisfaction following OCTR.	Retrospective design; no comparator group.
Petrover et al., 2017 [27]	Prospective cohort (UGCTR)	Levels II–III	129	6 months	Demonstrated feasibility with significant short‐term symptom and functional improvement following UGCTR.	Single‐center, nonrandomized study with limited long‐term follow‐up.
Cano et al., 2024 [51]	Prospective registry cohort	Levels II–III	102 patients (162 hands)	Up to 6 years	Sustained clinical improvement reported after ultrasound‐guided CTR; findings support continued evaluation in clinical practice.	Registry‐based study without randomized comparator.
Eberlin et al., 2024 [52]	Multicenter randomized trial (CTR‐US vs. mini‐OCTR)	Level I (RCT)	122	1 year	CTR‐US demonstrated comparable symptom and functional improvement to mini‐OCTR, with smaller incisions and reduced scar discomfort; long‐term equivalence remains unproven.	Limited to 1‐year follow‐up.
Emerging evidence (non–peer‐reviewed preprint)						
Ulusoy et al., 2025 [53]	Prospective comparative study (preprint; not peer‐reviewed)	Emerging Evidence (preprint)	172	Short‐ and long‐term assessments	Preliminary findings suggest faster recovery and fewer early complications with CTR‐US; results require confirmation in peer‐reviewed comparative trials.	Preprint (not peer‐reviewed); findings should be interpreted as preliminary and should not be accorded the same evidentiary weight as peer‐reviewed studies.

*Note:* EMG, electromyography; PROMs, patient‐reported outcome measures; CTR‐US, carpal tunnel release with ultrasound guidance.

Abbreviations: CTS, carpal tunnel syndrome; ECTR, endoscopic carpal tunnel release; mOCTR, mini‐open carpal tunnel release; NR, not reported; OCTR, open carpal tunnel release; PCTR, percutaneous carpal tunnel release; QoL, quality of life; RCT, randomized controlled trial; TCL, transverse carpal ligament.

^∗^Levels of evidence are based on study design. Studies presented under “Emerging Evidence” have not undergone peer review and are included to illustrate evolving developments in the field. Their findings should be interpreted with caution and were not accorded the same evidentiary weight as peer‐reviewed studies.

## 9. Comparative Analysis of Surgical Techniques

The quality of evidence supporting each surgical approach varies considerably. Evidence for OCTR and ECTR is supported by multiple RCTs, systematic reviews, meta‐analyses, and long‐term follow‐up studies, whereas evidence for ultrasound‐guided and other ultra‐minimally invasive techniques remains predominantly based on prospective cohorts, pilot studies, registry data, and cadaveric investigations. Consequently, conclusions regarding these newer techniques should be interpreted with appropriate caution.

### 9.1. Clinical Outcomes

#### 9.1.1. Symptom Relief

Across surgical approaches, the primary therapeutic goal remains complete division of the TCL to relieve median nerve compression. High‐quality randomized trials comparing OCTR and ECTR consistently demonstrate substantial and durable improvement in pain, nocturnal paresthesia, and numbness, with no clinically meaningful difference in long‐term symptom resolution beyond 6–12 months [40, 49].

Meta‐analyses of RCTs confirm that early symptom improvement may occur slightly faster following ECTR; however, pooled long‐term patient‐reported outcome measures (PROMs), including the BCTQ, show equivalence between OCTR and ECTR at final follow‐up [54].

Emerging UGCTR cohorts report rapid early symptom improvement, often within 2–4 weeks, but the majority of studies involve selected patients with mild‐to‐moderate disease and limited follow‐up durations (< 2 years) [40, 55]. Long‐term durability beyond 5 years remains insufficiently characterized.

#### 9.1.2. Functional Scores

Functional recovery is typically assessed using BCTQ functional status scales, DASH scores, and grip strength measurements. RCTs demonstrate that ECTR confers modest early functional advantages, including improved grip strength and earlier return to daily activities during the first 4–8 postoperative weeks [36, 49].

However, by 6–12 months, functional outcomes converge across techniques [54]. The magnitude of early grip strength improvement following ECTR, while statistically significant in pooled analyses, is often modest and may not reach clinically meaningful thresholds for all patients [54].

UGCTR studies report favorable early BCTQ and DASH improvements comparable to open or mini‐open approaches at 6–12 months [55]. Nevertheless, the absence of large multicenter RCTs limits definitive conclusions regarding long‐term equivalence.

#### 9.1.3. Recurrence Rates

Recurrence following carpal tunnel release is generally uncommon, with reported rates ranging from approximately 1%–10% across techniques [56]. Importantly, recurrence must be distinguished from persistent symptoms, which are typically attributable to incomplete TCL division or misdiagnosis.

Comparative studies suggest that recurrence is more closely related to adequacy of decompression and patient‐specific factors, such as diabetes, inflammatory conditions, or postoperative fibrosis, rather than the surgical approach itself [40, 56]. Current UGCTR data report low short‐term recurrence; however, robust long‐term reoperation data are limited [54].

### 9.2. Complication Profiles

#### 9.2.1. Nerve Injury

Major neurovascular injury remains rare across all techniques but differs mechanistically. Meta‐analytic data indicate a slightly increased relative risk of transient nerve injury with ECTR compared with OCTR, particularly during early adoption phases [54]. These injuries are most often neurapraxias rather than permanent deficits.

OCTR, while offering direct visualization, is associated with palmar cutaneous branch irritation and pillar pain. UGCTR theoretically reduces blind dissection through real‐time imaging, but operator inexperience may increase the risk of incomplete release or iatrogenic injury [55].

#### 9.2.2. Infection and Wound Complications

Superficial wound complications are more commonly reported with OCTR due to larger incisions [36]. ECTR and UGCTR demonstrate lower rates of scar tenderness and wound‐related morbidity in early postoperative periods. Deep infection rates are low and comparable across techniques.

#### 9.2.3. Incomplete Release

Incomplete division of the TCL is a leading cause of persistent symptoms. Early ECTR literature identified higher rates of incomplete release during the learning curve; contemporary series suggest improved rates with surgeon experience [54]. UGCTR also carries risk of incomplete release if sonographic landmarks are misinterpreted, underscoring the importance of structured training.

### 9.3. Recovery and Return to Work

Time to functional recovery represents one of the most clinically meaningful differentiators. Multiple RCTs demonstrate earlier return to work following ECTR compared with OCTR, particularly among manual laborers [36, 49]. Differences range from approximately 1–3 weeks in pooled analyses.

UGCTR cohorts report even shorter early recovery intervals in selected populations, with some studies documenting return to routine activities within 7–14 days [54]. However, these findings derive primarily from small or single‐center studies and require confirmation in large randomized comparisons.

From an occupational perspective, earlier functional restoration may significantly reduce indirect costs related to work absenteeism.

### 9.4. Cost‐Effectiveness

Cost analyses must account for both direct operative costs and indirect economic impact. OCTR generally requires minimal equipment and is associated with lower procedural costs. ECTR entails higher equipment and setup costs but may offset these through earlier return to productivity [57]. Formal cost‐effectiveness analyses suggest that when reduced work absenteeism is considered, ECTR may approach cost neutrality in employed populations [57].

Economic data for UGCTR remain limited. Although procedure time and facility requirements may be reduced, the need for high‐resolution ultrasound equipment and operator expertise influences cost modeling. Comprehensive health economic evaluations are needed before broad adoption.

### 9.5. Learning Curve and Surgeon Expertise

Surgeon experience plays a pivotal role in outcome optimization. OCTR is widely regarded as technically straightforward with a relatively short learning curve. ECTR requires dedicated training, and complication rates are higher during early adoption [49].

UGCTR introduces a dual skill requirement: surgical decompression principles and advanced musculoskeletal ultrasound proficiency. Variability in operator training remains a key barrier to standardization [55]. Ultimately, surgeon expertise may exert a greater influence on complication rates and completeness of release than the specific technique employed.

Across techniques, long‐term symptom relief and functional outcomes are largely equivalent when decompression is complete. ECTR offers consistent early recovery advantages, particularly regarding return to work and scar tenderness. UGCTR demonstrates promising early results but requires further high‐quality comparative trials with long‐term follow‐up.

Taken together, we interpret the accumulated evidence as indicating that the choice among OCTR, ECTR, and UGCTR is no longer primarily a question of comparative long‐term efficacy, since all three achieve equivalent symptom relief once decompression is complete; the clinically decisive variable is operator training and resource context rather than which technique is used. This reframes what we view as the central open question in the field: rather than continuing to ask whether one technique outperforms another in symptom resolution, future comparative research should instead define the specific training thresholds, sonographic landmark criteria, and standardized outcome‐reporting requirements needed to make UGCTR as procedurally reliable as OCTR and ECTR have become through decades of standardization. Until such benchmarks are established, we recommend that UGCTR be positioned as a selectively applied adjunct in appropriately resourced, high‐volume centers rather than as a default alternative, a distinction that existing systematic reviews and meta‐analyses, which largely pool heterogeneous UGCTR series without stratifying by operator experience, have not made explicit.

## 10. Patient Selection and Personalized Surgical Decision‐Making

The selection of surgical technique for CTS should be individualized, integrating disease severity, anatomical considerations, comorbid conditions, occupational demands, and surgeon expertise. Although long‐term clinical outcomes following OCTR and ECTR are broadly equivalent, meaningful differences in early recovery profiles and complication patterns influence patient‐specific procedural choice [58, 59].

OCTR remains the most dependable technique in complex clinical scenarios. It is particularly appropriate in revision surgery, where scar tissue or incomplete prior release necessitates wide exposure and direct visualization of the TCL and median nerve. Similarly, patients presenting with severe CTS characterized by thenar atrophy, significant axonal loss on electrodiagnostic studies, or suspected space‐occupying lesions may benefit from an open approach that allows comprehensive inspection of the carpal tunnel contents. Anatomical distortion secondary to trauma or prior surgery further supports the use of OCTR. Long‐term cohort data demonstrate durable symptom relief and low reoperation rates exceeding 10 years after OCTR, reinforcing its role as a reliable benchmark technique [50].

ECTR may offer advantages in carefully selected patients, particularly those prioritizing rapid functional recovery. RCTs consistently demonstrate earlier return to work and reduced postoperative scar tenderness following ECTR compared with OCTR, although long‐term symptom relief and functional outcomes remain comparable [58, 60]. Younger individuals, manual laborers, and patients seeking smaller incisions for cosmetic reasons may therefore benefit from endoscopic release when performed by experienced surgeons. However, surgeon proficiency remains critical, as complication rates during the early learning phase are higher.

UGCTR and other ultra‐minimally invasive techniques represent emerging options in selected populations. Current evidence suggests favorable short‐term symptom improvement and accelerated recovery in patients with mild‐to‐moderate CTS without significant anatomical variation [61, 62]. Nevertheless, these data are primarily derived from small prospective cohorts and limited randomized comparisons, and long‐term durability beyond 5 years remains insufficiently characterized. Accordingly, patient selection for UGCTR should be cautious and performed in centers with expertise in musculoskeletal ultrasound.

Comorbidities also influence procedural selection and postoperative recovery. Diabetes mellitus, obesity, inflammatory arthropathies, and hypothyroidism are associated with delayed wound healing and may increase postoperative complication risk [50]. In such populations, techniques minimizing soft tissue disruption may theoretically reduce wound‐related morbidity, although high‐quality comparative data are limited. Similarly, anticoagulation status and bleeding risk require individualized perioperative planning, although careful medical optimization often exerts a greater influence on outcomes than technique selection alone.

Occupational considerations are particularly relevant in working‐age patients. Earlier return to productivity following ECTR may offset higher operative costs in employed individuals, as suggested by cost‐effectiveness analyses [63]. Conversely, in retired or sedentary patients, early recovery differences may hold less practical significance. Ultimately, given the overall equivalence in long‐term outcomes across established techniques, shared decision‐making is essential. A transparent discussion of expected recovery timelines, complication risks, cosmetic considerations, and surgeon experience should guide individualized procedural selection. Key patient‐specific factors that may influence selection of the most appropriate surgical technique for CTS are summarized in Table 2.

**TABLE 2:** Patient‐specific factors influencing selection of the optimal surgical technique for carpal tunnel syndrome.

Patient factor	Preferred surgical technique	Rationale	Supporting evidence
Severe CTS with thenar atrophy or axonal loss	OCTR	Allows direct visualization and ensures complete TCL release in advanced disease	Kim et al. [13]; Louie et al. [50]
Revision CTS surgery	OCTR	Scar tissue and altered anatomy require open exposure	Botte et al. [34]
Anatomical variations (bifid nerve, persistent median artery)	OCTR	Safer visualization of neurovascular structures	Henry et al. [10]
Younger working patients needing rapid return to work	ECTR	Faster early functional recovery and reduced scar tenderness	Sayegh and Strauch [40]; Atroshi et al. [60]
Manual laborers requiring early functional restoration	ECTR	Earlier return to occupational activity	Brown et al. [49]; Agee et al. [36]
Cosmetic preference/scar minimization	ECTR or UGCTR	Smaller incision and improved cosmetic outcomes	Miles et al. [28]; Ekhtiari et al. [61]
Mild–moderate CTS without anatomical complexity	UGCTR	Percutaneous decompression with minimal tissue disruption	Petrover et al. [27]; Cano et al. [51]
Patients at risk of wound healing complications (diabetes, obesity)	ECTR or UGCTR	Reduced soft tissue disruption and smaller incision	Zimmerman et al. [21]; Ekhtiari et al. [61]
Anticoagulated patients or high bleeding risk	UGCTR (select cases)	Percutaneous technique with minimal dissection	Cognet et al. [43]
Surgeons early in training	OCTR	Short learning curve and predictable anatomy exposure	Munns and Awan [41]

Abbreviations: CTS, carpal tunnel syndrome; ECTR, endoscopic carpal tunnel release; OCTR, open carpal tunnel release; TCL, transverse carpal ligament; UGCTR, ultrasound‐guided carpal tunnel release.

## 11. Emerging Technologies and Future Directions

### 11.1. Current Evidence‐Supported Applications

Recent technological advances have expanded the role of digital innovations in the diagnosis, surgical planning, and perioperative management of CTS. Among these, artificial intelligence (AI) has shown the greatest potential in diagnostic support rather than direct surgical intervention. Machine learning algorithms have demonstrated promising accuracy in the interpretation of ultrasound images, electrodiagnostic studies, and clinical datasets, facilitating earlier diagnosis and improved patient stratification [62, 63]. However, despite these encouraging developments, the current evidence remains predominantly observational and algorithm‐validation–based, with limited prospective clinical studies demonstrating improved patient outcomes. Accordingly, AI should currently be regarded as an adjunctive diagnostic and decision‐support tool rather than an established component of routine surgical management.

### 11.2. Investigational and Future Technologies

Robotic‐assisted carpal tunnel release represents another emerging area of interest. Robotic platforms may offer theoretical advantages, including enhanced instrument precision, tremor reduction, and improved reproducibility of minimally invasive procedures [64, 65]. However, current evidence supporting robotic‐assisted carpal tunnel surgery is limited primarily to experimental investigations, cadaveric feasibility studies, engineering validation, and proof‐of‐concept reports. Clinical evidence evaluating patient outcomes, safety, cost‐effectiveness, and long‐term durability remains scarce. Consequently, robotic‐assisted carpal tunnel release should presently be regarded as an investigational technology rather than an established clinical treatment modality [66].

Other emerging innovations, including augmented reality, computer‐assisted navigation, advanced intraoperative imaging, and novel surgical instrumentation, may further enhance the precision of minimally invasive carpal tunnel release in the future.

## 12. Authors’ Clinical Synthesis and Practical Recommendations

The accumulated evidence indicates that contemporary surgical techniques for CTS should not be viewed as competing procedures with a universally superior option but rather as complementary approaches suited to different clinical scenarios. Across decades of comparative research, complete division of the TCL consistently emerges as the principal determinant of long‐term success, irrespective of the surgical approach employed.

From the authors’ perspective, OCTR should remain the preferred option for revision surgery, severe CTS with thenar atrophy, space‐occupying lesions, uncertain anatomy, and situations where maximal visualization is required. Although ECTR offers measurable advantages in early postoperative recovery, these benefits primarily affect the first few postoperative weeks and become less clinically meaningful over longer follow‐up, during which functional outcomes converge with OCTR.

Ultrasound‐guided techniques represent an exciting evolution toward tissue‐preserving surgery; however, current evidence remains insufficient to support routine replacement of established approaches. Their adoption should presently be limited to experienced centers with expertise in musculoskeletal ultrasound and carefully selected patients without complex anatomy.

Importantly, surgeon expertise appears to influence outcomes more than the choice of surgical approach itself. Numerous reported complications, particularly incomplete release and transient nerve injury, are associated with the learning curve rather than inherent procedural limitations. Consequently, surgeons should preferentially perform the technique with which they possess the greatest experience while individualizing treatment according to patient expectations, occupational requirements, anatomical considerations, and available resources.

Based on the current evidence, the authors propose that future comparative research should move beyond determining whether one technique is superior to another. Instead, studies should focus on identifying which patients benefit most from each surgical strategy, incorporating standardized patient‐reported outcomes, cost‐effectiveness analyses, and long‐term follow‐up.

## 13. Limitations of the Review

This review has several limitations that should be acknowledged. First, as a structured narrative review, it was not conducted as a formal systematic review and therefore did not include protocol registration or duplicate independent study selection; risk of bias and quality were nonetheless formally assessed for the key comparative studies using design‐appropriate tools (Cochrane RoB 2, AMSTAR‐2, and the NOS; Supporting Table S1), although these appraisals were performed by the primary reviewers with consensus discussion rather than by independent duplicate assessment.

Second, the available literature is heterogeneous with respect to study design, patient selection, surgical techniques, outcome measures, and duration of follow‐up, limiting direct comparisons across studies. Third, although every effort was made to incorporate the most recent evidence, much of the literature on ultrasound‐guided and other ultra‐minimally invasive techniques consists of pilot studies, small prospective cohorts, registry‐based reports, and emerging investigations with limited long‐term follow‐up. Consequently, conclusions regarding these newer techniques should be interpreted cautiously and considered within the context of the current evidence base.

## 14. Conclusion

Surgical management of CTS has evolved from traditional open release to endoscopic and, more recently, ultra‐minimally invasive ultrasound‐guided techniques. Current evidence indicates that when complete decompression of the TCL is achieved, OCTR and ECTR provide comparable long‐term clinical outcomes, while endoscopic techniques may offer advantages in early postoperative recovery. Emerging ultrasound‐guided techniques show encouraging short‐term results but continue to require robust long‐term comparative evidence before their role can be fully established.

Rather than supporting a universally superior surgical approach, the available evidence favors individualized technique selection based on disease severity, patient characteristics, anatomical considerations, surgeon expertise, and available resources. Future research should focus on adequately powered multicenter randomized trials, standardized outcome reporting, long‐term follow‐up, and cost‐effectiveness analyses to better define the role of emerging ultra‐minimally invasive techniques.

## Author Contributions

Nikeeta Nancoo, Ira Bhasin, and Nasir Kharma: writing–original draft, methodology, and data collection; Yousef Al Shahomi, Amar Shamsah, and Ezzeldin A. Shalaby: writing–original draft, methodology, and visualization; Amanda Jane Abel, Aaron Samuel, Helal Salem Alhajri, Najeeb Ansari, and Bramaes Dahal: methodology, visualization, and data collection; Manju Rai: supervision and writing–review and editing.

## Funding

No funding or sponsorship was received for this study or publication of this article.

## Disclosure

All the authors approved the final version to be published.

## Conflicts of Interest

The authors declare no conflicts of interest.

## Supporting Information

Additional supporting information can be found online in the Supporting Information section.

## Supporting information


**Supporting Information** Supporting File S1: Provides risk of bias and quality assessment of key comparative studies cited in Table 1.

## Data Availability

All data generated or analyzed during this study are included in this published article.
