# Supplementary material for: Advancements in Surgical Management of Carpal Tunnel Syndrome: A Comparative Review of Open, Endoscopic, and Ultra‐Minimally Invasive Techniques
Source: Adv Orthop. 2026 Aug 31;2026:9652002. doi: 10.1155/aort/9652002 (PMC13528443; doi:10.1155/aort/9652002)
Supplement: Supplementary file 1 [file AORT-2026-9652002-s001.docx]

## **Supplementary Table S1**

**Table S1: Risk of Bias and Quality Assessment of Key Comparative Studies Cited in Table 1, Using Design-Appropriate Validated Instruments (Cochrane RoB 2 for randomized controlled trials; AMSTAR-2 for systematic reviews/meta-analyses; Newcastle-Ottawa Scale for non-randomized cohort/registry studies).**

| **Study (Author, Year)** | **Study Design** | **Appraisal Tool Used** | **Key Domain-Level Observations** | **Overall Rating** |
| --- | --- | --- | --- | --- |
| Agee et al., 1992 [36] | Multicenter RCT (ECTR vs OCTR) | Cochrane RoB 2 | Randomization process: low risk (multicenter, randomized). Outcome measurement: some concerns due to reliance on early-generation endoscopic technology and limited long-term PROMs. | Some concerns |
| Brown et al., 1993 [52] | Prospective RCT (ECTR vs OCTR) | Cochrane RoB 2 | Randomization process: low risk. Missing outcome data/measurement of outcome: some concerns due to short (12-week) follow-up and early endoscopic instrumentation. | Some concerns |
| Eberlin et al., 2024 [50] | Multicenter RCT (CTR-US vs mini-OCTR) | Cochrane RoB 2 | Randomization, deviations from intended intervention, and outcome measurement: low risk. Applicability limited by 1-year follow-up horizon (noted as an indirectness concern, not a bias domain). | Low risk |
| Sayegh & Strauch, 2015 [40] | Meta-analysis of RCTs | AMSTAR-2 | Protocol and search strategy adequately reported; critical weakness in a priori registration not confirmed. Heterogeneity among pooled trials and variability in surgeon experience limit certainty of pooled estimates. | Moderate confidence |
| Vasiliadis et al., 2014 [42] | Cochrane systematic review | AMSTAR-2 | Comprehensive search strategy, duplicate study selection/data extraction, and pre-registered protocol (Cochrane methodology). Variable methodological quality flagged among primary studies included in the review. | High confidence |
| Louie et al., 2013 [59] | Retrospective cohort (no comparator) | Newcastle-Ottawa Scale | Selection domain downgraded: no concurrent comparator group and single-arm design. Outcome ascertainment and follow-up adequacy: satisfactory (>10-year follow-up, low loss to follow-up). | Fair (5/9 stars) |
| Petrover et al., 2017 [27] | Prospective cohort (UGCTR) | Newcastle-Ottawa Scale | Selection and comparability domains downgraded: single-center, nonrandomized, no comparator arm. Outcome domain adequate (validated functional scores, defined follow-up). | Fair (5/9 stars) |
| Cano et al., 2024 [49] | Prospective registry cohort | Newcastle-Ottawa Scale | Selection domain downgraded: registry-based recruitment, no randomized comparator. Outcome domain adequate (long follow-up up to 6 years, objective outcome assessment). | Fair (5/9 stars) |
| Ulusoy et al., 2025 [51] | Prospective comparative study (preprint; not peer-reviewed) | Not formally appraised (peer-review status precludes standard tool application) | Non-peer-reviewed status introduces indeterminate risk of reporting and publication bias; methodological details not yet independently verified. | High risk / Indeterminate |

*Abbreviations: RCT = Randomized Controlled Trial; ECTR = Endoscopic Carpal Tunnel Release; OCTR = Open Carpal Tunnel Release; CTR-US = Carpal Tunnel Release with Ultrasound Guidance; mOCTR = Mini-Open Carpal Tunnel Release; UGCTR = Ultrasound-Guided Carpal Tunnel Release; RoB 2 = Cochrane Risk of Bias tool, version 2; AMSTAR-2 = A MeaSurement Tool to Assess systematic Reviews, version 2; PROMs = Patient-Reported Outcome Measures.*
